# Mixed Cation Halide Perovskite under Environmental and Physical Stress

**DOI:** 10.3390/ma14143954

**Published:** 2021-07-15

**Authors:** Rosanna Larciprete, Antonio Agresti, Sara Pescetelli, Hanna Pazniak, Andrea Liedl, Paolo Lacovig, Daniel Lizzit, Ezequiel Tosi, Silvano Lizzit, Aldo Di Carlo

**Affiliations:** 1 CNR-Institute for Complex Systems, Via dei Taurini 19, 00185 Rome, Italy; 2 C.H.O.S.E. (Centre for Hybrid and Organic Solar Energy), Department of Electronic Engineering, University of Rome Tor Vergata, 00133 Rome, Italy; aldo.dicarlo@uniroma2.it; 3 Institut Pprime, UPR 3346 CNRS, Université de Poitiers, ISAE-ENSMA, BP 30179, 86962 Futuroscope-Chasseneuil, France; poznyak.a87@gmail.com; 4 INFN-LNF, Via Enrico Fermi 54, 00044 Rome, Italy; andrea.liedl@lnf.infn.it; 5 Elettra-Sincrotrone Trieste S.C.p.A., AREA Science Park, S.S. 14 km 163.5, 34149 Trieste, Italy; paolo.lacovig@elettra.eu (P.L.); ezequiel.tosi@elettra.eu (E.T.); silvano.lizzit@elettra.eu (S.L.); 6 LASE—Laboratory of Advanced Solar Energy, National University of Science and Technology “MISiS”, Leninsky Prospect 4, 119049 Moscow, Russia

**Keywords:** mixed perovskite, perovskite stability, MXene, XPS, work function

## Abstract

Despite the ideal performance demonstrated by mixed perovskite materials when used as active layers in photovoltaic devices, the factor which still hampers their use in real life remains the poor stability of their physico-chemical and functional properties when submitted to prolonged permanence in atmosphere, exposure to light and/or to moderately high temperature. We used high resolution photoelectron spectroscopy to compare the chemical state of triple cation, double halide Cs${}_{x}$(FA${}_{0.83}$MA${}_{0.17}$)${}_{\left(1-x\right)}$Pb(I${}_{0.83}$Br${}_{0.17}$)${}_{3}$ perovskite thin films being freshly deposited or kept for one month in the dark or in the light in environmental conditions. Important deviations from the nominal composition were found in the samples aged in the dark, which, however, did not show evident signs of oxidation and basically preserved their own electronic structures. Ageing in the light determined a dramatic material deterioration with heavily perturbed chemical composition also due to reactions of the perovskite components with surface contaminants, promoted by the exposure to visible radiation. We also investigated the implications that 2D MXene flakes, recently identified as effective perovskite additive to improve solar cell efficiency, might have on the labile resilience of the material to external agents. Our results exclude any deleterious MXene influence on the perovskite stability and, actually, might evidence a mild stabilizing effect for the fresh samples, which, if doped, exhibited a lower deviation from the expected stoichiometry with respect to the undoped sample. The evolution of the undoped perovskites under thermal stress was studied by heating the samples in UHV while monitoring in real time, simultaneously, the behaviour of four representative material elements. Moreover, we could reveal the occurrence of fast changes induced in the fresh material by the photon beam as well as the enhanced decomposition triggered by the concurrent X-ray irradiation and thermal heating.

## 1. Introduction

The combination of optimal electronic properties and ease of processability makes metal halide perovskites the most promising material for the fabrication of next-generation optoelectronic devices. In photovoltaic the ideal band gap for solar light absorption, the optimal absorption coefficients and charge carrier lifetime have led to solar cell efficiency as high as 25.5% [1]. On the other hand the relatively simple processing techniques make possible to tune the electronic properties through the material stoichiometry, by simply adjusting the composition of the precursor solutions. This last aspect has dramatically widened the scientific activity on the synthesis and characterization of simple and more complex perovskites, strongly increasing in a short time the know-how about this class of materials and promoting an unprecedented improvement of the conversion efficiency of perovskite based solar cells (PSCs). In the last few years, the interest has moved from simple to mixed perovskites. These latter systems can be in general represented as AMX${}_{3}$, where A is a mixture of the organic (methylammonium MA CH${}_{3}$NH${}_{3}^{+}$; formamidinium FA CH${}_{3}$(NH${}_{2}$)${}_{2}^{+}$, guanidinium CH${}_{6}$N${}_{3}^{+}$, acetamidinium CH${}_{3}$C(NH${}_{2}$)${}_{2}^{+}$), and inorganic (Cs${}^{+}$, Rb${}^{+}$) cations, M is the Pb${}^{2+}$ ion and X is a mixture of halides (I, Br, Cl). Optimal performance in solar cells was obtained by combining a triple cation MA/FA/Cs configuration with a mixture of I and Br halides, in solutions of FAPbI${}_{3}$ and MAPbBr${}_{3}$ [2]. However, both for simpler and more complex perovskites, the issue that still hampers the PSC commercial applicability and that is drawing a great deal of the scientific attention is the long-term stability of the materials when exposed to unavoidable physical and chemical stresses [3,4]. In comparison with the iconic MAPbI${}_{3}$ perovskite exploited in PSCs, a higher resilience to lattice decomposition, coupled with improved conversion performance was pursued by substituting, wholly or partly, MA with the more stable FA and I with Br, being this latter choice motivated by the stronger bond of the more electronegative halide to the perovskite lattice [5]. However, if on one side combining I and Br was reported to favour a higher long-term stability in comparison to single halide perovskites [2], on the other side the coexistence of the two halides in the lattice generates phase segregation under illumination [6,7,8,9,10], resulting in intrinsic material doping and poor device operational stability. Then the implemented perovskites, although featuring superior conversion efficiency, are also prone to complex electronic and structural instabilities when interacting with external agents. Such intriguing scenario asks for deep investigations to elucidate the occurrence and the severity of the different degradation mechanisms. In recent years, several studies on the stability of triple cation mixed halide perovskites were carried out, mostly focused on the halide segregation under photo-excitation [11,12] or electrical bias [13], due to the direct implications of these effects in device efficiency and long term performance. On the contrary, plain environmental ageing and thermal induced decomposition have been less frequently investigated [14], although the evolution of the fundamental material properties in simple contexts is preliminary to the understanding of more complex processes.

In this study, we used high resolution (HR) X-ray photoelectron spectroscopy (XPS) to carry out an extensive characterization of triple cations (MA, FA and Cs) double halides (I and Br) perovskite Cs${}_{x}$(FA${}_{0.83}$MA${}_{0.17}$)${}_{\left(1-x\right)}$Pb(I${}_{0.83}$Br${}_{0.17}$)${}_{3}$ (in the following indicated as PV) thin films, with the primary objective to compare the chemical state of fresh PV layers with that of similar films exposed to prolonged environmental ageing while kept in the dark or in the light. Our second aim was to explore the effect that a particular class of ethero-nanomaterials used as dispersed additive to the precursor solution might have on the PV resistance to different degradation agents. In fact, if on one side the addition of 2D nanomaterials as transition metal dichalcogenides [15,16], graphene [17,18], graphene oxide [19] and other carbon-based materials [20] has been reported to improve the PSC efficiency, on the other side the concurrent stability improvement is not taken for granted. As an example, when graphene oxide and related materials [21] are used as interlayer in PSCs, reactive oxygen species can be produced under prolonged illumination eventually triggering the photodegradation of perovskite. In the present work, we focussed our attention on the 2D titanium carbide Ti${}_{3}$C${}_{2}$T${}_{x}$ MXene flakes, where T${}_{x}$ indicate the functional groups (-OH, =O, -F) which terminate the flakes after etching procedures or post-processing [22]. Such choice being motivated by the superior performance observed in PSCs based on PV with added Ti${}_{3}$C${}_{2}$T${}_{x}$ flakes [23,24]. Our objective was to exclude any detrimental effect of the dispersed MXene flakes on the intrinsic PV behaviour and hopefully reveal possible beneficial influence on the material stability. We pursued this objective by preparing PV layers containing dispersed MXene flakes and carrying out on them the same ageing procedure done on the undoped layers. In the last part of this study we investigated the action of the thermal stress on the chemical state of PV layers in order to identify the sequence of reactions leading to material decomposition. While heating the PV samples, we used fast-XPS to monitor simultaneously the evolution of different PV component elements. Working in ultra-high vacuum (UHV) allowed us to exclude the interaction with chemical agents as moisture and oxygen and observe the mere effect of physical stresses. Moreover, along the whole study, we carefully considered the possible presence of unwanted X-ray photoinduced effects on the material chemistry. Considering the fragile chemical stability of perovskites, the awareness that in some conditions, the measured spectra might exhibit spurious information due to the interaction with the photon beam is of primary importance when dealing with a widely used characterization method as XPS [25,26,27,28], especially when high intensity synchrotron radiation is used as photon source.

## 2. Results and Discussion

Cs${}_{x}$(FA${}_{0.83}$MA${}_{0.17}$)${}_{\left(1-x\right)}$Pb(I${}_{0.83}$Br${}_{0.17}$)${}_{3}$ (PV) layers and Ti${}_{3}$C${}_{2}$T${}_{x}$ MXene doped PV (in the following indicated as PVMX) layers, were deposited on fluorine-doped tin oxide (FTO) coated glass substrates and characterized after 30 days by HR-XPS. During this time, a first batch of samples was kept in vacuum and in the dark and was then considered fresh. Two other batches, maintained in air with controlled humidity (30%HR), were continuously kept in the dark or also exposed to 1-sun light for 150 h in total, and were thus considered “dark-aged” and “light-aged”, respectively.

### 2.1. Fresh Samples

**Figure 1:**
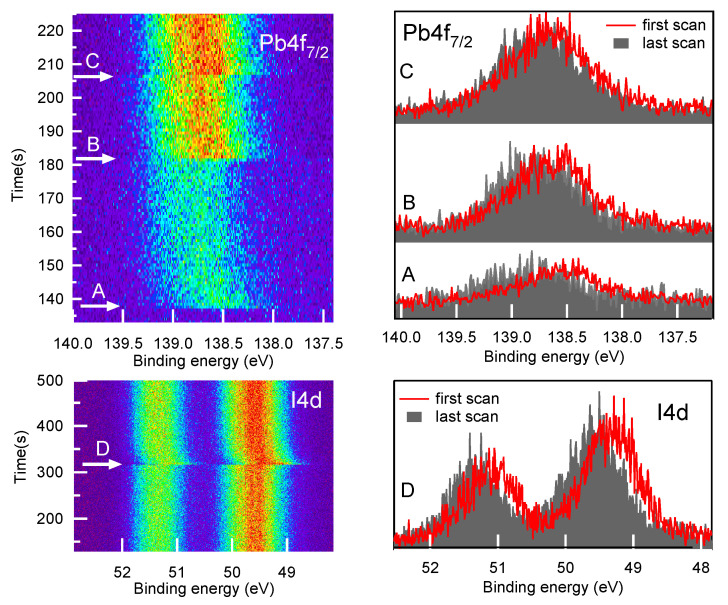
X-ray photon-induced core level shifts. (**Left**) 2D map of Pb4f${}_{7/2}$ spectra (top, acquisition time of each spectrum t${}_{acq}$ = 0.5 s) and I4d spectra (bottom, t${}_{acq}$ = 1.0 s) acquired in “snapshot acquisition mode” at photon energy of 520 eV and plotted as a function of time (*y* axis). The changes of the sample point in the sequence are marked by the arrows. (**Right**) Pb4f${}_{7/2}$ and I3d first scan (red) and last scan (grey, solid) measured each time in a new sample point (A, B, C and D) never irradiated before. The different signal intensity in the different points is mainly due to the loss of alignment while moving the sample rapidly far away from the positions where it had been optimally aligned.

Before starting the measurement runs on the fresh samples, we verified the occurrence of possible changes in the material, due to the direct action of the X-ray photons or to the interaction with photoelectrons and secondary electrons. Therefore, we collected fresh PV samples core level spectra in fast modality, using the so-called “snapshot acquisition mode” (see Experimental Section). The results obtained for the I4d and Pb4f${}_{7/2}$ spectra are displayed in Figure 1. The photon beam (hν = 520 eV) was fixed in the same sample point and then shifted to a new point in correspondence of the arrows. It is clear that in less than 10 s the peaks shifted by 100 ÷ 250 meV as a result of the severe electronic perturbation induced by the X-ray beam, and after that their BE positions remained stable (see Figure S1). The shift was observed each time that the beam hit a sample point never irradiated before. The same behaviour was also observed for PVMX samples. The sensitivity of pure- [29] and mixed-halide perovskites [30] to visible and even γ radiation [31] has been often reported. In mixed halides, perovskite photo-excitation was proven to determine a reversible phase separation into iodine-rich and bromide-rich domains [30], recently attributed to the local strain generated by the interaction of the photo-excited charges with the ionic lattice [32]. In our study, in order to further investigate the fast dynamics of the photon beam induced effects and to establish the reversibility of the electronic perturbation, a dedicated time-resolved diagnostics would have been needed. However, the mere description of what we observed might entice the scientific community to investigate the effects of X-ray excitation in perovskite materials and to clarify whether the observed core level shifts are also referable to fast charge-carrier transport and ion motion effects. Considering that in all sample points the binding energy (BE) position of a certain peak converged to the same value and that after several minutes without X-ray beam exposure, we did not observe any inverse BE shift, which would indicate a recovery of the initial state, in the following we take as representative for our samples the stable final BE positions. It is also worth noting that without the “snapshot acquisition mode” we would have not been aware of the fast changes that are absolutely undetectable when acquiring the XPS spectra in the standard way, i.e., in the so-called “scan modality” which requires much longer acquisition times (see Experimental Section).

**Figure 2:**
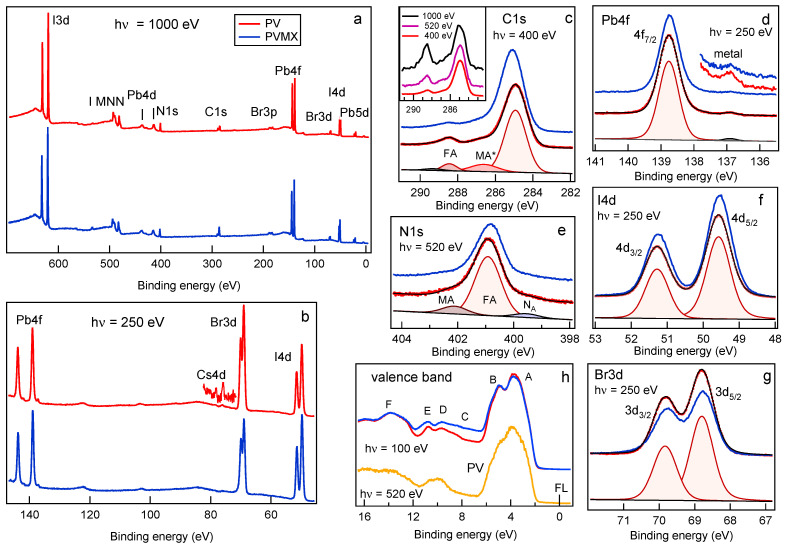
XPS characterization of freshly deposited PV (red curves) and PVMX (blue curves) samples. (**a**) Bulk sensitive (hν = 1000 eV) and (**b**) surface sensitive (hν = 250 eV) survey spectra normalized to the intensity of the Pd4f doublet; (**c**) C1s (hν = 400 eV), (**d**) Pb4f${}_{7/2}$ (hν = 250 eV), (**e**) N1s (hν = 520 eV), (**f**) I4d (hν = 250 eV) and (**g**) Br3d (hν = 250 eV) core level spectra. In each case the PV spectra are shown together with the best-fit curves (black) and the spectral components. In the C1s spectrum the component MA* includes the contribution of the MA cation plus that of surface contaminants. (**h**) Valence band spectra measured at photon energy of 100 eV on PV and PVMX samples; for PV the spectrum taken at 520 eV is also shown.

**Figure 3:**
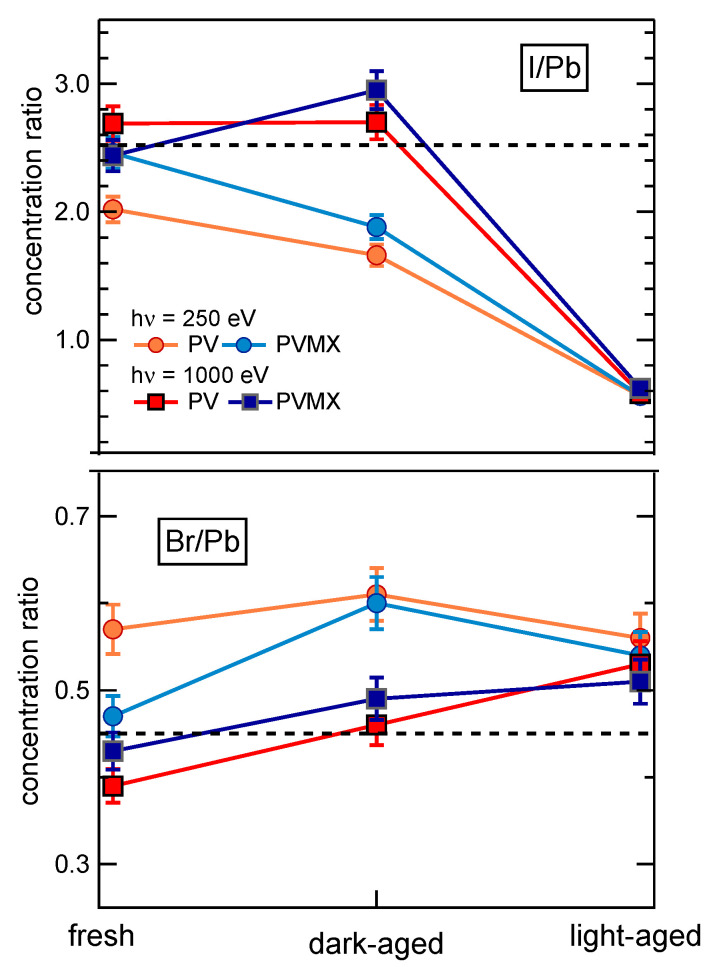
(**Top**) I/Pb and (**bottom**) Br/Pb concentration ratios obtained from the XPS spectra measured for fresh, dark-aged and light-aged PV and PVMX samples (compare Table S2). The lines between adjacent points are only a guide for the eyes. The dashed lines indicate the expected I/Pb and Br/Pb nominal values calculated by considering the layer as having the same composition of the precursor solution (i.e., including the tiny PbI${}_{2}$ excess, see Experimental Section).The error bars are deduced from the results of the characterization in different points of at least two samples of each category.

Figure 2a,b shows the comparison between the XPS survey spectra measured at photon energy of 1000 eV and 250 eV on fresh PV (red curves) and PVMX (blue curves) samples. The photon energies were selected to enhance the bulk (hν = 1000 eV) or the surface (hν = 250 eV) sensitivity of the XPS technique, so as to have the possibility to compare the chemical composition of the samples at the surface and in the layers underneath. The spectra clearly show the Pb, I, Br, C and N core levels, as well as the tiny Cs 4d doublet. In the spectrum measured at 1000 eV the I MNN Auger feature also appears around 490 eV. In none of the PVMX spectra, features arising from the Ti${}_{3}$C${}_{2}$T${}_{x}$ additive could be detected, in agreement with the low quantity contained in the doped samples. The ratios among the Pb, Br and I concentrations at the surface (hν = 250 eV) and in a thicker perovskite layer (hν = 1000 eV) were obtained by weighting the Pb4f, Br3d and I4d integrated intensity with the corresponding photoionzation cross sections at each photon energy [33] (see Table S1). The confidence of such evaluation is ensured by the fact that the three selected core levels are all located within a BE range of ∼100 eV, which implies that the corresponding photoelectrons come predominantly from similar depths of the order of 6–7 Å and 10–15 Å at 250 and 1000 eV, respectively. The Pb:I:Br ratios obtained for the fresh PV and PVMX samples are listed in Table S2 and the I4d/Pb4f and Br3d/Pb4f normalized intensity ratios are plotted in Figure 3.

For PV, the surface-sensitive spectrum shows mild I depletion and Br excess with respect to the nominal composition, which is better approached in the bulk sensitive spectrum, where, however, the Br and I differences with respect to the expected concentrations are inverted.

As will be discussed below, such a discrepancy likely reflects the occurrence of halide migration even in the fresh samples. On the contrary, for PVMX the Pb:I:Br ratios at the surface and along the layer depth, more closely agree with the nominal composition expected from the precursors concentrations. From these results, it seems that the presence of Ti${}_{3}$C${}_{2}$T${}_{x}$ flakes in the material decreases the halide migration rate and keeps for a longer time the nominal stoichiometry also at the film surface. As for the Pb:N ratio, we found values close to 1:1.7 in both samples, at the surface (XPS spectra measured at hν = 520 eV, see Figure S2) and in the bulk, whereas the correct quantification of the C abundance in PV and PVMX is hindered by the contribution of the surface contaminants. In the high resolution spectra shown in Figure 2c–g, for both PV and PVMX samples the Br3d and I4d spectra exhibit a single doublet component, whereas the Pb4f spectrum, in addition to the main peak due to Pb in the PV lattice, exhibits the tiny feature fingerprint of metallic Pb. The BE positions found by best-fitting the XPS spectra are reported for each element in Table 1, which lists also the BE values measured for the aged samples. The MA and FA cations are represented by the C1s and N1s spectra displayed in Figure 2c,e, respectively. In the N1s spectrum, the intense and the weak components are attributed in order to the ammonium NH${}_{3}^{+}$ and amine NH${}_{2}$ functional groups [34,35,36] and are indicative of the FA and MA ligands, respectively.

**Figure 4:**
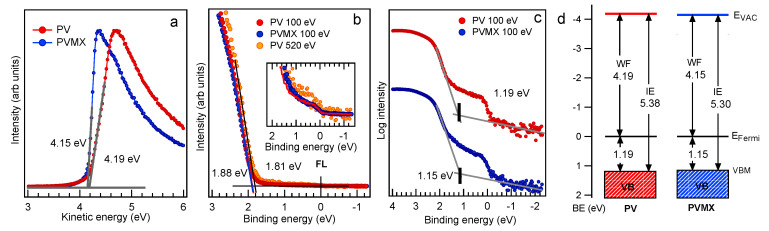
(**a**) Secondary electron cutoff (SECO) and (**b**,**c**) valence band maximum (VBM) measured for fresh PV and PVMX samples. The VBM are plotted in linear scale in (**b**) and in logarithmic scale in (**c**). The inset in (**b**) shows the Fermi level region plotted with an expanded vertical scale. (**d**) Energy scheme for PV and PVMX with respect to E${}_{Fermi}$. (IE, ionization energy. E${}_{VAC}$, vacuum level.)

The ratio of ∼8 measured between their integrated intensities is somewhat lower than that (∼9.8) expected from the FA/MA nominal ratio. The additional tiny N${}_{A}$ peak arises from dissociated organic phases (see below). The C1s spectra of both samples are dominated by the intense peak at ∼285 eV due to C-C and C-H bonds in surface contaminants and shows the MA and FA components [37], whose relative intensities rise with the photon energy (see inset of Figure 2c), as the contribution of the surface contaminants becomes less important with increasing probe depth. Interestingly, in the C1s spectra measured at 1000 eV the ratio between the FA and MA components approaches the nominal value of 5. This indicates that at the surface the MA peak, which appears more intense than the FA component, also contains spurious contributions due to contaminants (and therefore is indicated in Figure 2 as MA *). The PV and PVMX valence band spectra are displayed in Figure 2h. The assignment of the A-F spectral features taken from Ref. [37] are reported in the Supplementary Materials. Here it is worth focussing the attention on the valence band maxima (VBM), which are plotted together with the positions of the secondary electrons cutoff (SECO) in Figure 4. The VBM extrapolated from the curves plotted in linear scale in Figure 4b is found to be at 1.88 eV for both samples (1.81 eV if measured with 520 eV photons). The magnification of the Fermi level region displayed in the inset in Figure 4b shows the presence of a tiny intensity in correspondence of the Fermi level, due to the minimal fraction of metallic Pb. When using the logarithmic intensity plot to extract the VBM (see Figure 4c), values shifted by ∼−0.7 eV are obtained [37], in closer agreement with the VBM values of 1.31–1.36 eV [14,38] reported in the literature for a mixed perovskite layer with the composition similar [14] or slightly different [38] from that of our samples. Here, as in many other perovskite materials, as was shown in Ref. [39], due to the low density of states at the top of the valence band, the VBM extracted from the linear plot underestimates the onset, which is more appropriately extrapolated from the logarithmic intensity plot. Therefore the energy scheme displayed in Figure 4d includes the VBM values extrapolated in Figure 4c.

The PV and PVMX work functions (WFs) are very similar to each other, resulting 4.19 ± 0.05 eV and 4.15 ± 0.05 eV, respectively, with a negligible influence of the Ti${}_{3}$C${}_{2}$T${}_{x}$ dopant. In this case, the WF measured on the fresh MXene batch used to dope the PVMX sample was 4.35 eV (see Figure S4). In contrast, a measurable WF tuning due to the Ti${}_{3}$C${}_{2}$T${}_{x}$ addition was reported in Ref. [23] for MAPbI${}_{3}$ and triple cation perovskite, with the PV (MAPbI${}_{3}$) work function decreasing from 4.72 (4.73) eV to 4.37 (4.62) eV due to the effect of the lower WF (3.69 eV) of the MXene dopant used in that preparation. In fact, for Ti${}_{3}$C${}_{2}$T${}_{x}$ the WF is closely related to the distribution of the O/OH/F flake terminations [40], which drive the charge transfer between the MXene and the perovskite lattice [23].

### 2.2. Aged Samples

#### 2.2.1. Samples Kept in the Dark

**Figure 5:**
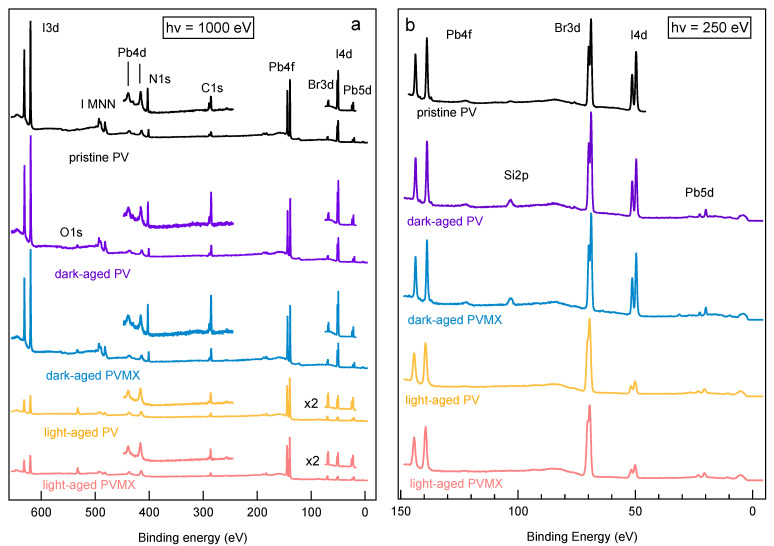
Survey spectra measured with photon energy of (**a**) 1000 eV and (**b**) 250 eV on PV and PVMX samples kept for 30 days in air with controlled humidity (30%HR) in the dark (“dark-aged”) or also exposed to solar radiation (“light-aged”). The corresponding spectra measured on a fresh PV layer (see Figure 2) are also shown for comparison at the top of the panels. The spectra are normalized to the integrated intensity of the Pb4f doublet.

Figure 5a,b show the XPS survey spectra measured on “dark aged” and “light aged” PV and PVMX samples at photon energies of 1000 and 250 eV, respectively. The effects of the dark-ageing are similar in PV (violet curves) and PVMX (blue curves).

The Pb(I${}_{x}$Br${}_{y}$)${}_{3}$ compositions reported in Table S2 as derived from the survey spectra taken at 250 eV and 1000 eV indicate that the permanence in air, even without exposure to light, has moderately depleted of I and enriched with Br the surface of both samples. Interestingly, Figure 3 well shows that the I depletion and Br excess revealed at the sample surface disappear in the layer underneath since in the bulk sensitive spectrum the Br3d/Pb4f and I4d/Pb4f ratios approach the nominal value. The observed deviations from the nominal perovskite stoichiometry indicates the migration of Br${}^{-}$ ions into iodide vacancies at the film surface. Iodine transport away from the surface of MAPbI${}_{3}$ layers exposed to laser beams [41] and halide migration in mixed perovskites under intense light stresses [30] have been often observed and related to the action of electric field or local strains [32] photogenerated in the perovskite material. The dark-aged samples have been only exposed to the ambient light during layer growth and for the time needed for the insertion in the UHV chamber for the XPS measurements. Though a similar exposure to ambient light was also withstood by the fresh samples, for the dark-aged samples the asymmetry between the I and Br content at the surface and in the layer underneath appears much more pronounced, and similar for PV and PVMX samples. These results lead to the conclusion that mixed halide perovskites are, to some extent, intrinsically prone to halide migration, even when not exposed to light. According to Figure 5, after the dark-ageing the higher C1s intensity signals the increased quantity of surface contaminants and the appearance of the O1s peak indicates the adsorption of water molecules.

**Figure 6:**
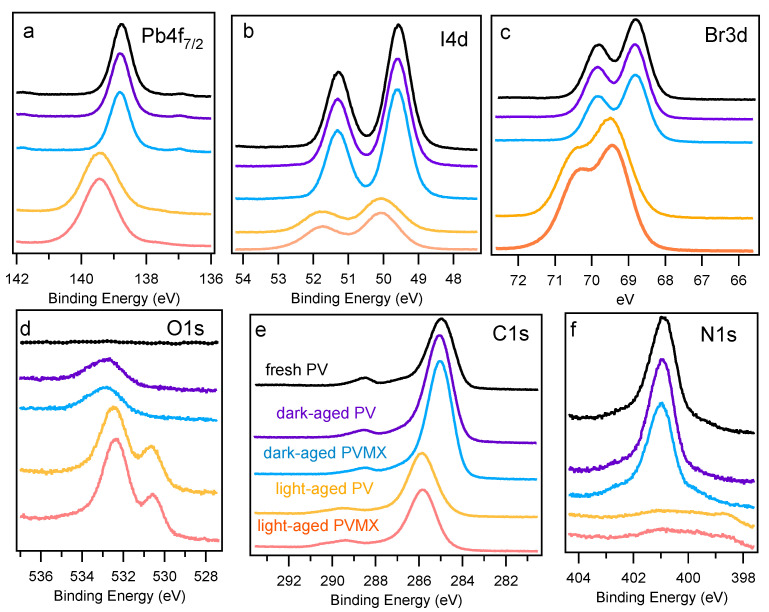
High resolution XPS spectra measured on PV and PVMX samples kept for 30 days in air with controlled humidity (30%HR) in the dark (“dark-aged”) or also exposed to solar radiation (“light-aged”): (**a**–**c**) Pb4f${}_{7/2}$, I4d and Br3d (hν = 250 eV), (**d**) O1s (hν = 650 eV); (**e**) C1s (hν = 400 eV), (**f**) N1s ((hν = 520 eV). The corresponding spectra measured on fresh PV (compare Figure 2) are shown also for comparison at the top of the panels.

The high resolution spectra shown in Figure 6 indicate that the Pb4f, I4d, and Br3d features remain almost unchanged with respect to the fresh samples, due to a substantial chemical stability of the perovskite phase, when kept in the dark.

**Figure 7:**
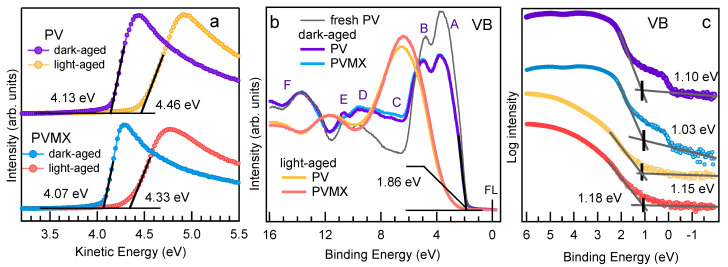
(**a**)Secondary electron cutoff, (**b**) valence band spectra and (**c**) valence band maxima (plotted in logarithmic scale) measured with hν = 100 eV for dark-aged and light-aged PV and PVMX samples. In (**b**) the VB spectrum measured on fresh PV is also shown (black line) (compare Figure 2h)).

Likewise, the low quantity of oxygen, together with the lack of any evident oxidized component in the high resolution spectra of the different elements, attests to the resilience to oxidation of the perovskite materials not heavily exposed to light. The observed O1s spectrum can be attributed mainly to adsorbed water molecules, whereas the intensity increase of the main C1s component is also mainly due to contaminant adsorption. The valence band spectra measured in the dark-aged samples (see Figure 7b) manifest a reduction of the spectral weight between 2 and 6 eV with respect to the fresh PV sample The reduction is more pronounced at the VB top where the I-Pb states emit [37], in agreement with the surface depletion of I revealed by the core level spectra. Both for PV and PVMX the VBM derived from the linear plot remain similar to those of the fresh samples, whereas shifts of −90 meV and −120 meV for PV and PVMX, respectively, are extracted from the curves plotted in the logarithmic scale (see Figure 7c). In this case the comparison with Figure 4c confirms that the amount of metallic Pb, responsible for the intensity at the Fermi level, is comparable with the quantity detected in the fresh samples. The curves measured in the SECO region (see Figure 7a) indicate that the WFs, which are 4.13 and 4.07 for PV and PVMX, respectively, are only slightly lower than those of their fresh counterparts (compare Figure 4). The evidence that both VBM and WF values decrease with respect to the fresh samples might be due to the combined effect of adsorbed water molecules [42] and some forms of doping resulting from the altered Pb:I:Br ratios.

#### 2.2.2. Samples Kept in the Light

**Figure 8:**
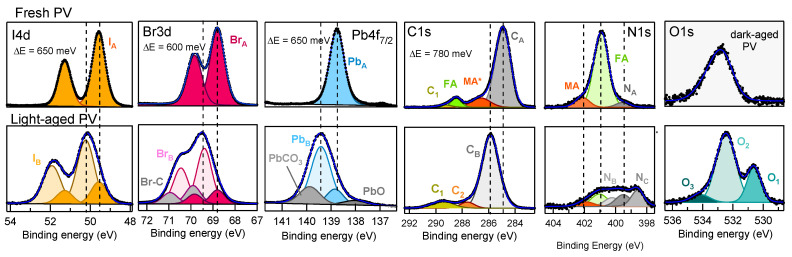
Decomposition of the high resolution core level spectra measured on fresh (top row; compare Figure 1) and light-aged (bottom row; compare Figure 6) PV samples. In the C1s spectrum measured on the fresh sample, the component MA* includes the contribution of the MA cation plus that of surface contaminants. Only for O1s do the top panel show the spectrum measured for the dark-aged PV sample since the corresponding spectrum measured on the fresh sample was nearly flat. The ΔE value in each top panel indicates the shift between the BE positions of the X${}_{A}$ and X${}_{B}$ (X = C, I, Br, Pb) components marked by the vertical dashed lines. The BE positions of the spectral components are listed in Table 1.

Much stronger changes are observed for the samples exposed to light. The survey spectra shown in Figure 5a,b show that also in this case do the PV (yellow curves) and the PVMX (orange curves) samples undergo equivalent modifications irrespective for the presence of the MXene additive. In comparison to the fresh and dark-aged samples, now the Pb:I:Br ratios are completely altered both in the surface-sensitive and in the bulk-sensitive spectra, where the I3d and I4d doublets are reduced to less of one third (compare Table S2). The high resolution core level spectra shown in Figure 6f reveal a strong loss of N. Moreover, in comparison with the dark-aged samples, the O1s spectra are much more intense and deeply modified, and the Pb4f${}_{7/2}$, I4d and Br3d spectral shapes are broader and appear blue-shifted by several hundreds meV. Due to the applicative relevance of the chemical effects photoinduced in these materials by the visible radiation, the core level spectra of the light-aged samples were analysed in detail to unravel the effects leading to the modification of the spectral line shapes. The results obtained by best-fitting the PV spectra are summarized in Figure 8 where also the corresponding fresh spectra are shown for comparison (compare Figure 2). The considerations reported in the following for these samples can be extended also to the doped PVMX samples which, as evident in Figure 5 and Figure 6, exhibit nearly identical spectral line shapes. The BE positions of the spectral components are listed in Table 1. For the Pb4f${}_{7/2}$, Br3d and I4d spectra we found some residual intensity of the fresh PV spectra (components Pb${}_{A}$, I${}_{A}$, Br${}_{A}$) and, in each case, intense new components (Pb${}_{B}$, I${}_{B}$, Br${}_{B}$) shifted by 600-650 meV to higher BE. The stability of the spectra under the X-ray beam, even after prolonged irradiation, excludes that the shifts are due to charging effects. On the basis of the discussion reported in the Supplementary Materials we can conclude that the appearance of these new components is a consequence of the electric field photogenerated by the X-ray beam in the degraded perovskite matrix, which determines blue-shifted spectral features.

This effect cannot be clearly disentangled from the severe chemical degradation and the possible presence of doping. The leftover of the original spectra (Pb${}_{A}$, I${}_{A}$ and Br${}_{A}$ components) likely arises from sample domains, where degradation is less pronounced. This last assumption is confirmed by the curves measured in the N1s regions (see Figure 6f), which exhibit weak intensity between 401 and 399 eV, and, as shown in Figure 8, can be decomposed into components due to residual FA ad MA phases and to their dissociated fragments (N${}_{A}$, N${}_{B}$ and N${}_{C}$). Interestingly, the N1s spectra are not shifted to higher BE as is observed for all other elements, which likely indicates that the residual N-containing phases are mainly located in the less degraded PV domains, from where the surviving Pb${}_{A}$, I${}_{A}$ and Br${}_{A}$ components are supposed to arise. In the heavily deteriorated regions, the organic phases are deeply dissociated and converted into volatile products within light-catalysed surface reactions with adsorbed molecules. Although the substitution of I with Br should provide a higher stability to the PV lattice [5], in these samples the ageing process is so advanced that both (FA/MA)PbI${}_{3}$ and (FA/MA)PbBr${}_{3}$ phases are extensively decomposed, which makes impossible the identification of any possible difference between their photolability. In contrast to the dark-aged samples, in this case the similar I:Br ratios measured at the sample surface and in the bulk (see Figure 3 and Table S2) excludes that the observed iodine depletion is caused by I migration away from the illuminated surface followed by the inverse Br transport, unless we hypothesize that the migration occurs on a length more extended than the depth probed by XPS. More reasonably, the intense loss effectively signals I desorption, likely as HI, whereas bromine remains in the layer where it also binds to organic fragments, as indicated by the Br-C component [43,44] appearing the in the Br3d spectrum in Figure 8. As for Pb, in addition to Pb${}_{A}$ and Pb${}_{B}$ we identify the formation of PbO and PbCO${}_{3}$ [45,46,47,48] (details for the assignment are given in the Supplementary Materials). In the O1s spectrum, the components O${}_{1}$, O${}_{2}$ and O${}_{3}$ appearing after light-ageing can be mainly related to C-O/PbO, C=O and O-C=O bonds in surface contaminants and in oxidized organic and inorganic phases. The same bonds are predominantly responsible for the weak C${}_{1}$ and C${}_{2}$ components of the C1s spectrum that after light ageing mainly substitute the contribution of the MA and FA cations now heavily decomposed. As in the fresh samples, the main C1s peak, now strongly blue-shifted, arises from the C-C and C-H bonds in contaminants and in fragments of the organic PV phase. As for the presence of PbI${}_{2}$, due to the similar chemical environment of the Pb${}^{2+}$ and I${}^{-}$ ions in the perovskite matrix and in PbI${}_{2}$, the I and Pb core level spectra are degenerate for the two materials, so that they cannot be distinguished by XPS. Therefore we cannot exclude that PbI${}_{2}$ forms in the aged layers, but the minimal quantity of iodine and the presence of PbO and PbCO${}_{3}$ likely indicate that in the decomposed material the Pb detached from the perovskite matrix is mostly bound to C and O rather to I atoms. The absence of residual metallic Pb, is proven by the lack of any edge at the Fermi level in the curves plotted in Figure 7c.

The valence bands measured on PV and PVMX at photon energies between 100 and 1000 eV are displayed in Figure 7b and in Figure S3. The spectra measured at hν≥ 250 eV are nearly overlapping one another, and their profile, modified with respect to the less aged samples, comes out from the altered chemical composition, with a depletion of the Pb-I band at the top of the VB [37], associated with the ∼650 meV up-shift discussed above for the core level spectra. Instead, the VB spectra measured with 100 eV photons, which are mostly sensitive to the surface layer, when plotted in linear scale (see Figure 7b), show a leading edge blue shifted by ∼2 eV with respect to the spectra taken on less aged samples. However from the plot in logarithmic scale (Figure 7c) it can be deduced that the VBM edge, although broadened, is comparable with that of the fresh samples, which suggests that the minimal intensity at the top of the VB arises from the less aged domains originating the I${}_{A}$, Br${}_{A}$ and Pb${}_{A}$ components in the corresponding core level spectra (see Figure 8). In the SECO region, likely for the presence of oxidized phases, both for PV and PVMX samples the cutoff is ∼0.3 eV higher than in the dark-aged samples. Congruent VBM and SECO shifts keeping an almost stable ionization potential are expected in the case of plain doping, band bending and photovoltage effects [49,50,51]. For the degraded PV and PVMX materials, the asymmetry in the VBM (shifted to higher BE) and SECO (shifted to higher kinetic energy) variations confirms that the origin of the spectral shifts is a complex superposition of intrinsic and external effects.

### 2.3. Thermal Stress

In the last part of this study, we investigated the behaviour of freshly deposited samples under thermal stress. Due to the negligible effect of the MXene doping on the PV aging observed so far, neither for the PV reaction to thermal stress we expected large variation related to the presence of the additives and therefore restricted this experiment to the undoped PV samples. We followed simultaneously the N1s, Pb4f, I4d and Br3d spectra while heating the sample, to reveal changes in the concentration and in the chemical state of the different elements. Moreover, in order to highlight possible X-ray beam induced effects, we carried out the experiment twice, once by keeping the beam hitting the sample continuously while increasing the temperature and the second time by keeping the X-ray beam shuttered, except during the short time interval (70 s in total) needed to measure fast N1s, Pb4f, I4d and Br3d spectra at selected temperatures.

**Figure 9:**
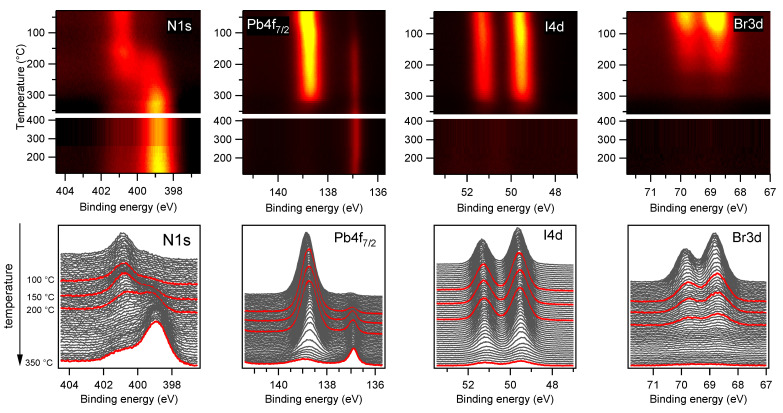
Thermal annealing of the PV sample under X-ray irradiation. (Top row) 2D plots of the N1s, Pb4f${}_{7/2}$, I4d and Br3d spectra measured while heating the sample with a linear temperature ramp and during the subsequent cooling to RT. The individual spectra measured during sample heating are displayed in the bottom row panels, vertically shifted for clarity. The red curves highlight the spectra measured at the selected temperatures indicated on the left side of the figure.

**Figure 10:**
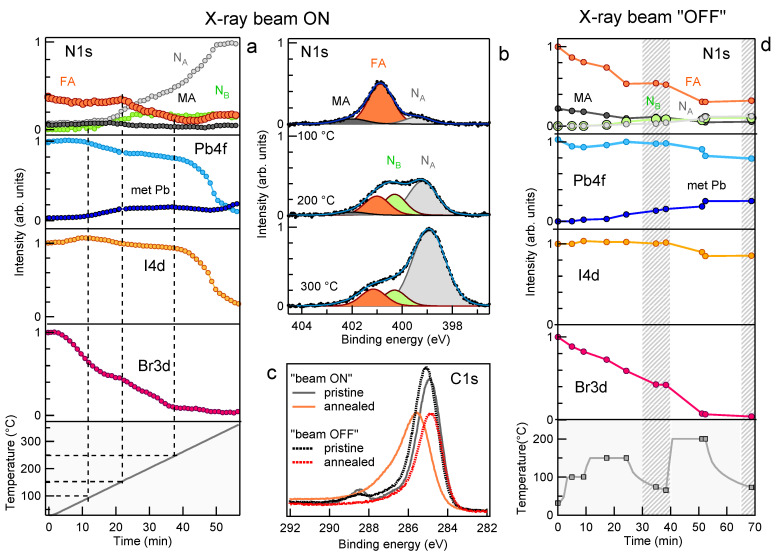
PV chemical composition under thermal stress. (**a**,**d**) Integrated intensity of the N1s, Pb4f, I4d and Br3d spectra measured at photon energy of 520 eV during the thermal annealing of the PV sample (**a**) with the X-ray beam continuously ON (see Figure 9) and (**d**) with the X-ray beam mostly OFF (see Figure 11) plotted as a function of the annealing time. The bottom panel shows in each case the corresponding sample temperature. The decomposition of the N1s spectra measured at 100, 200 and 300 ${}^{\circ }$C during the first experiment is shown in (**b**). Analogous N1s spectral decomposition was carried out for the other experiment and the results are reported in the top graph of panel d. (**c**) Comparison among the C1s spectra measured on the fresh samples, and on the samples annealed with the X-ray beam continuously ON and mostly OFF.

The results of the first experiment are summarized in Figure 9. The upper panels display the 2D images obtained by plotting the series of the N1s, Pb4f${}_{7/2}$, I4d and Br3d spectra acquired during the thermal annealing and during the subsequent cooling to RT as a function of the sample temperature reported on the vertical axis. The individual core level spectra, vertically shifted for a better visualization, are shown in the bottom panels. The spectra sequences were best fitted using, in each case, but not for N1s, the same components shown in Figure 2, as no spectral features due to the formation of new chemical bonds were detected. The component intensities, reported in Figure 10a, provide the evolution of the PV composition during thermal annealing. The most remarkable findings emerging in Figure 10a are the dramatic drop of the Br3d intensity, which starts to decay around 50 ${}^{\circ }$C and continues to decrease with the annealing temperature up to its complete extinction, and the behaviour of the N1s spectrum, which shows a strong increase of the overall intensity together with a marked line shape modification. By examining in more detail the curves plotted in Figure 10a (see also Figure S5) it turns out that between RT and 85 ${}^{\circ }$C the Br3d and I4d intensity decreases by 25% and increases by 7%, respectively. It seems then that the Br loss triggers the I migration towards the surface, thus leading to a higher I4d signal. The dip appearing in the FA curve between 50 and 150 ${}^{\circ }$C (see Figure S5), which is the opposite of the hill shape in the I curve, likely manifests the changing screening of the N atoms due to substitution of Br${}^{-}$ with bigger I${}^{-}$ ions. If the Br loss is due to desorption and/or to in-diffusion to a depth greater than that probed by XPS cannot be established.

The consistent Br depletion observed at low temperature is at odd with a recent study on a similar mixed perovskite layer reporting the complete stability of the Br concentration during prolonged heating at 85 ${}^{\circ }$C [14]. A possible way to reconcile such contrasting results is to assume the occurrence of anticipated perovskite photo-dissociation due to X-ray irradiation simultaneous with thermal heating. The evolution of the organic cations is in principle well traced by the N1s spectrum (see Figure 10a,b). However, the onset of MA and FA dissociation is not plainly monitored by the intensity of the corresponding components, since as reported above, these are sensitive to the changing screening consequent to the halides displacement. Anyway, from the curves plotted in Figure 10a and Figure S5, it can be certainly stated that MA and FA are unstable above 135 and 155 ${}^{\circ }$C, respectively, and that their manifest decay is concomitant with the strong increase of the N${}_{A}$ component and with the appearance of the new component N${}_{B}$. The presence in the N1s spectrum of photoemitted intensity below 401 eV has been often observed during MAPbI${}_{3}$ degradation and attributed to the progressive deprotonation of the methylammonium [34,35]. In particular, peaks at ∼400 eV and below 400 eV have been attributed to methylamine [34,52] or Pb amide [35] and to free amine [35], respectively. Accordingly, the N${}_{A}$ and N${}_{B}$ peaks can be related to the decomposition of the organic cations, with the formation of -NH${}_{2}$ and possibly (-NH) functional groups. Then, differently from what occurred in the light-aged samples, which were almost N-free, in this case the NH${}_{x}$ fragments remain in the solid phase. It is possible that polymerization reactions, with the formation of new N-C bonds are triggered directly by the continuous X-ray photon flux, and, more probably, by the low-energy secondary electrons generated in the material. The formation of new N-C bonds is suggested also by the modified C1s line shape measured in the annealed sample (see Figure 10c). PV decomposition generates PbI${}_{2}$ and metallic Pb phases, the latter remanining below 20% up to 250 ${}^{\circ }$C. Above this temperature the Pb4f and I4d peaks decay quite abruptly together while shifting and broadening, thus revealing the massive PbI${}_{2}$ dissociation. In parallel the N1s intensity reaches twice the initial value, due to a lower and lower screening provided by the other PV components which have progressively desorbed.

**Figure 11:**
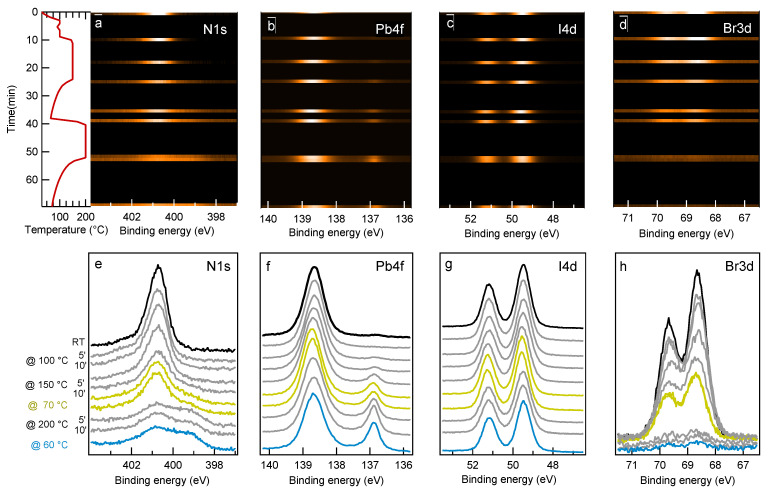
Thermal annealing of the PV sample with the X-ray beam mostly OFF. (Top row) 2D plots of the N1s, Pb4f${}_{7/2}$, I4d and Br3d spectra measured during sample heating. The vertical axis reports the annealing time whereas the sample temperature is represented by the curve on the very left side of the top row. The X-ray beam was shuttered for most of the time and opened only for the acquisition of the set of fast spectra, which appear as bright lines. After heating to 150 ${}^{\circ }$C the sample was let to cool down to RT to monitor eventual changes in the chemical composition of the sample. Bottom row) Individual N1s, Pb4f${}_{7/2}$, I4d and Br3d spectra measured during heating, vertically shifted for clarity. The yellow (cyan) spectra were measured during the intermediate (after the final) cooling.

Figure 11 summarizes the results obtained by annealing the PV sample with the photon beam almost always shuttered. As reported above, in this case the sample was exposed to the X-ray radiation only for the time necessary to acquire fast N1s, Pb4f, I3d and Br4d spectra every ∼5 min, as illustrated by the 2D images in Figure 11a–d. In the mean while the sample temperature was not raised with a linear ramp but increased in steps, each one lasting about 10 min as shown in the left panel of Figure 11a. Moreover we also investigated a possible recovery of the perovskite structure after the relieve of the thermal stress by letting the sample cool down after having been heated to 150 and 200 ${}^{\circ }$C. The spectra displayed in Figure 11e–h show that even when strongly limiting the X-ray dose, the PV reaction to heating remains severe. However, the analysis of the spectra sequence reported in Figure 10d illustrates that after 5 min at 100 ${}^{\circ }$C the Br loss was less than 20% (∼25% after 5 min at 150 ${}^{\circ }$C). Moreover, the PV sample heated at 150 ${}^{\circ }$C shows still the full of content of I and Pb, the latter being partially (∼15%) in the metallic phase. As for the MA and FA cations, both of them show some intensity decrease already after the first annealing step (5 min at 100 ${}^{\circ }$C), the depletion remaining however moderate up to 150 ${}^{\circ }$C. In comparison with the previous experiment, in this case the smaller Br loss and the lower iodine migration make the FA and MA intensity curves more adherent to the effective cation content and therefore a direct comparison between the two experiments could be misleading. Instead, some conclusion can be more reliably inferred for the inorganic components. The moderately higher stability observed for Pb as well as for both halides, validates the hypothesis that the stronger effect observed in the previous experiment was mainly photoinduced. This means that the combined photochemical and thermal excitation enhances the dissociation rate of the Br bonds with the PV lattice, which remains more stable when heated at moderate temperature (∼100 ${}^{\circ }$C) in the dark [14].

A similar reaction rate enhancement due to the combined photon and thermal excitations was observed while monitoring by XPS the heating of a MAPbI${}_{3}$ sample, which afterwords showed a much higher concentration of metallic lead in the X-ray beam spot than in the surrounding surface region [53]. To conclude the analysis of Figure 10d it is worth noting that no change in the element concentrations was observed during the intermediate cooling.

In view of the outcome of the two experiments the behaviour of PV under thermal annealing can be summarized by underlining that with concurrent continuous X-ray beam irradiation, heating at 50 ${}^{\circ }$C is enough to activate lattice decomposition, which is signaled by the Br decrease from the layer thickness probed by XPS. For the un-irradiated sample, our results show that a short (5 min) heating at 100 ${}^{\circ }$C seems sufficient for the onset of Br loss. Bromide desorption as BrH or Br${}_{2}$ then should be the most probable route [54]. Alternatively Br could drift toward the inside of the PV layer and accumulate at the interface with the substrate. Differently from Br, iodine is retained in the solid phase by the formation of PbI${}_{2}$. The mere comparison with the literature, which reports the MAPbI${}_{3}$ decomposition to occur around 85 ${}^{\circ }$C [4,55] would lead us to conclude that the partial substitution of I with Br the mixed perovskite, does not gain substantial lattice stabilization at moderately high temperature [56]. With and without continuous X-ray irradiation during heating the overall content of Pb stays constant up to 200–250 ${}^{\circ }$C, with not more than one-fifth segregated in the metallic phase. At variance with the samples light-aged in air, the fresh samples thermally stressed in UHV, even in the case of heavy degradation, did not show BE shifts in the Pb4f, I4d and Br3d spectra which could be taken as indicative for doping, Fermi level pinning or photovoltage effects. A discriminating factor between the two experiments is undoubtedly the absence in UHV of O${}_{2}$ and water molecules prone to adsorb and modify the surface electronic structure. Moreover, due to the lack of oxidizing agents prone to break the local Pb–I octahedral structure [57], the I content remains roughly constant in the solid phase. On the contrary, during light-ageing in atmosphere the oxidative reactions which consume Pb facilitate I desorption leading to a Br-rich material.

## 3. Materials and Methods

### 3.1. Perovskite Layer Deposition

After a deep cleaning sequence, consisting of triplicate steps of ultrasonic bath with cleaning liquid dissolved in deionized water, acetone and 2—propanol for 10 min each one, fluorine—doped tin oxide (FTO) coated glasses were transferred to a N${}_{2}$–filled glove box where the perovskite layer was deposited. A mix of FAI (1 M), PbI${}_{2}$ (1.1 M), MABr (0.2 M), PbBr${}_{2}$ (0.2 M) and CsI (0.075 M) in the mixture of anhydrous DMF/DMSO (4:1 vol/vol) was stirred at room temperature for 30 min to obtain the perovskite precursor solution. The solution was spin coated onto the samples with a one–step deposition and antisolvent method, consisting of a two-step program at 1000 and 5000 rpm for 10 s and 30 s respectively. During the second step, 200 μL of CB was poured on the spinning substrate 7 s prior to the end of the program. Immediately after spin coating, the substrates were annealed at 100 ${}^{\circ }$C for 1 h to form the perovskite crystal structure and obtain the PV samples. According to the amount of the compounds included in the precursor solution the nominal composition of the deposited layers, which will be compared with the XPS results, is Cs${}_{0.058}$(FA${}_{0.77}$MA${}_{0.15}$)Pb(I${}_{0.84}$Br${}_{0.15}$)${}_{3}$. However, along the text we consider the PV material to have the Cs${}_{x}$(FA${}_{0.83}$MA${}_{0.17}$)${}_{\left(1-x\right)}$Pb(I${}_{0.83}$Br${}_{0.17}$)${}_{3}$ composition by assuming that the excess of PbI${}_{2}$ mostly remains as is in the material, and leave undefined the Cs concentration since we cannot evaluate the percentage effectively substituting FA or MA in the perovskite lattice. The same perovskite precursor solution, doped by substituting 20% in vol with MXENE ink, was used in the same way, to fabricate the PVMX samples. The synthesis of MXene flakes from tha MAX phase and the preparation of the MXene ink are described in Ref. [23]. The amount of MXene which was added to the material corresponded to 0.014 mg in 1 ml of perovskite precursor solution. A first batch of deposited PV and PVMX layers was kept at low-vacuum (10${}^{-3}$ mbar) in containers immersed in an isolated environment (N${}_{2}$-filled glove-box system) and in the dark for 30 days and therefore were considered fresh samples. Two other batches were used for dark- and light-ageing. Therefore the samples, kept for 30 days in air, with controlled humidity (30%HR) and temperature (RT) conditions, were held in the dark and only the second also exposed to 1-sun light for 150 h (1-sun is the common measurement unit for irradiance equal to 1000 W/m${}^{2}$ or 100 MW/cm${}^{2}$). Fresh and aged samples were then introduced in individual parafilm-sealed boxes filled with nitrogen, and transferred to Elettra.

### 3.2. XPS Measurements

HR-XPS measurements were carried out at the SuperESCA beamline of the synchrotron radiation facility Elettra (Trieste, Italy). The photoemission spectra were measured with a Phoibos 150 mm electron energy analyser from SPECS Gmbh equipped with an home-made delay line detector. For each sample the sealed box was opened just before mounting it on the manipulator holder. Then the sample was rapidly introduced in the UHV (background pressure 1 × 10${}^{-10}$ mbar) analysis chamber through a fast entry lock. XPS measurements were carried out on the as-received samples, without performing any cleaning procedure. The measurements were carried out with the photon beam impinging at grazing incidence (70${}^{\circ }$), while photoelectrons were collected at normal emission angle. For the fast collection of the XPS spectra, with acquisition times that could be as low as 100 ms per spectrum, instead of running the analyser in the standard scanning mode we used the “snapshot acquisition mode”. In this case a complete spectrum is obtained by measuring in one shot and for a certain acquisition time t${}_{acq}$, the outcome of the ∼700 virtual channels of the delay–line detector along the energy dispersion direction, without scanning the lens voltage system of the analyser. The XPS survey spectra and the HR core level spectra were measured at the selected photon energies indicated in the text, with overall energy resolution of <300 meV for the measurements at h ν≥ 1000 eV and <100 meV for all the measurements performed at lower photon energies. For the temperature dependent measurements, 520 eV photons were used to acquire in sequence N1s, Pf4f, I4d, and Br3d core level spectra while heating the samples with a thermal ramp or increasing the temperature in steps. The temperature was measured by a thermocouple in good contact with the sample. Radiative heating through filaments placed behind the Mo plate holding the samples was used for the annealing. All spectra were calibrated versus the Fermi level at zero binding energy measured on a clean Au sample mounted in contact with the sample. Work functions were extracted with a linear extrapolation from the secondary electron cutoff. For these measured a bias of 8 V was applied to the sample. The error on the derived WF values is ±0.05 meV.

## 4. Conclusions

In this study, we used high resolution ultraviolet and X-ray photoelectron spectroscopy to investigate the plain environmental ageing and the thermal induced decomposition of the Cs${}_{x}$(FA${}_{0.83}$MA${}_{0.17}$)${}_{\left(1-x\right)}$Pb(I${}_{0.83}$Br${}_{0.17}$)${}_{3}$ mixed halide perovskite. We reached the following conclusions:

(i) the fresh samples show some deviation from the nominal composition since the surface and the layers underneath are found to be moderately I-poor and I-rich, respectively. The lattice truncation at the surface, which intrinsically implies chemical and electronic defects, seems to weaken the material stability so that even under negligible chemical and physical perturbations, the system rebalances by modifying the halide distribution along the layer depth.

(ii) This trend is accentuated after prolonged dark ageing in air. In this case, the more marked deviation from the nominal I/Br ratio together with the adsorption of water molecules leads to little modifications of the electronic structure at the PV surface, which, however, does not show signs of oxidation.

(iii) After the exposure to 1-sun light the material appears severely deteriorated, as nitrogen, and in large part also iodine, have been removed from a 1–2 nm thick near surface layer and Pb has in part reacted with contaminants.

Along this study we also investigated the possible effects of the Ti${}_{3}$C${}_{2}$T${}_{x}$ MXenes additive on the PV stability. We could exclude any detrimental influence on the PV stability, but also any measurable stabilizing effect of the added MXenes flakes on the long term ageing. Interestingly, in the fresh samples we revealed a moderate reduction of the initial rate of halide migration, which anyhow requires a more extensive verification.

Finally, we used fast-XPS to follow the surface reaction occurring in fresh PV sample during thermal annealing. In this case the PV decomposition initiates below 100 ${}^{\circ }$C and is signaled by the decrease of the Br content in the probed layer, accompanied by changes in the N1s spectrum. Differently from the samples aged in air, whose surface appears I poor, thermal heating in UHV removes Br much more than I. A possible explanation is related to contaminating molecules as O${}_{2}$, CO${}_{2}$ and H${}_{2}$O, which when present react with Pb, whereas their absence in UHV leaves PbI${}_{2}$ stable in the solid phase up to 250 ${}^{\circ }$C.

Our results confirm that XPS is an incomparable technique [25] to determine how the effective chemical composition of mixed perovskites deviates from the designed configuration and to reveal with high precision how the modification consequent to chemical or physical stresses propagates in the near surface layer. However, we must signal that the interaction between X-ray photons and mixed perovskites can alter the electronic state (and when coupled with thermal heating also the chemical state) of the materials, and that these phenomena, whose extent should be evaluated in each case, when using intense X-ray beams develop on a time scale of a few seconds.

To summarize, we can argue that our results on Cs${}_{x}$(FA${}_{0.83}$MA${}_{0.17}$)${}_{\left(1-x\right)}$Pb(I${}_{0.83}$Br${}_{0.17}$)${}_{3}$ demonstrate the existence of a serious criticalities concerning the stability of some mixed perovskites, which might overcome those of single halide materials. It seems then that the effective use of mixed perovskite for long-term applications appears dramatically conditioned by the success of remedies based on chemical and/or electronic stabilization of their functional properties and by the innovative design of device architectures capable of minimizing the interaction with perturbative agents.

## Figures and Tables

**Table 1:** Binding energies and assignments of the core level spectra measured for perovskite component elements and contaminants. For all core level doublets it is reported the energy position of the low BE component. The spin orbit splittings for Pb4f, I4d and Br3d core levels are 4.86, 1.70 and 1.04 eV, respectively. The component names reported in parenthesis are those defined in Figure 8. * marks the shifted BE values measured in the light-aged samples (see text); *n.i.* indicates that the component might be present with a very low intensity but cannot be identified in the spectrum.

Core Level	Assignment	Fresh	Dark-Aged	Light-Aged
		(eV)	(eV)	(eV)
Pb4f	PV (Pb${}_{A}$)	138.76	138.76	139.41 *(Pb${}_{B}$)
	metallic Pb	136.90	136.90	–
	PbO	–	–	138.04 *
	PbCO${}_{3}$	–	–	139.87 *
I4d	PV (I${}_{A}$)	49.57	49.57	50.22 * (I${}_{B}$)
Br3d	PV (Br${}_{A}$)	68.80	68.80	69.38 * (Br${}_{B}$)
	Br-C	–	–	69.87 *
N1s	FA	400.92	400.92	401.03
	MA	402.11	402.11	401.90
	dissociated organic phase, N${}_{A}$	399.55	399.55	399.50
	dissociated organic phase, N${}_{B}$	–	–	400.22
	dissociated organic phase, N${}_{C}$	–	–	398.58
C1s	C-C, C-H (C${}_{A}$)	284.92	284.92	285.87 * (C${}_{B}$)
	MA	286,60	286.60	*n.i.*
	FA	288,45	288.45	*n.i.*
	C=O, O=C–O (C${}_{1}$)	–	–	289.49 *
	C–O, PbCO${}_{3}$ (C${}_{2}$)	–	–	287.61 *
O1s	adsorbed water	–	532.80	–
	C–O, PbO (O${}_{1}$)	–	–	530.59 *
	C=O, PbCO${}_{3}$ (O${}_{2}$)	–	–	532.46 *
	O=C–O (O${}_{3}$)	–	–	534.11 *

## Data Availability

The data presented in this study are available on request from the corresponding author.

## Supplementary Materials

The following are available at https://www.mdpi.com/article/10.3390/ma14143954/s1, Table S1: Atomic photoionization cross sections; Figure S1: X-ray induced Pb4f${}_{7/2}$ and I4d${}_{5/2}$ core level shifts; Table S2: chemical composition of PV and PVMX samples in the various aging stages; Figure S2: survey spectra measured for dark-aged and light-aged PV and PVMX samples at 520 eV; Figure S3: Valence band and core level spectra measured on fresh and light-aged PV samples at different photon energies; Figure S4: Ti${}_{3}$C${}_{2}$T${}_{x}$ work function; Figure S5: Core level spectra intensity vs. temperature measured during PV sample annealing
