# Supplementary material for: Mixed Cation Halide Perovskite under Environmental and Physical Stress
Source: Materials (Basel). 2021 Jul 15;14(14):3954. doi: 10.3390/ma14143954 (PMC8303190; doi:10.3390/ma14143954)
Supplement: Supplementary file 1 [file materials-14-03954-s001.zip › materials-1250155-SI.pdf]

Mixed cation halide perovskite under environmental and physical stress
Supplementary Materials

Rosanna Larciprete,[*,†] Antonio Agresti,[*,‡] Sara Pescetelli,[*,‡] Hanna Pazniak,[¶] Andrea Liedl,[§] Paolo Lacovig,[‖] Daniel Lizzit,[⊥] Ezequiel Tosi,[‖] Silvano Lizzit,[‖] and Aldo Di Carlo[#]

†*CNR-Institute for Complex Systems, Via dei Taurini 19, 00185 Roma, Italy*

‡*C.H.O.S.E.(Centre for Hybrid and Organic Solar Energy), Department of Electronic Engineering, University of Rome Tor Vergata, Rome, Italy*

¶*Institut Pprime, UPR 3346 CNRS, Université de Poitiers, ISAE-ENSMA, BP 30179, 86962 Futuroscope-Chasseneuil Cedex, France*

§*INFN-LNF, Via Enrico Fermi 54, 00044 Frascati (Rome), Italy*

‖*Elettra-Sincrotrone Trieste S.C.p.A., AREA Science Park, S.S. 14 km 163.5, 34149 Basovizza (Trieste), Italy*

⊥*Elettra-Sincrotrone Trieste S.C.p.A., AREA Science Park, S.S. 14 km 163.5, 34149 Basovizza (Trieste), Italy; Current address: Polytechnic Department of Engineering and Architecture – D.P.I.A. University of Udine, 33100 Udine, Italy*

#*C.H.O.S.E.(Centre for Hybrid and Organic Solar Energy), Department of Electronic Engineering, University of Rome Tor Vergata, Rome, Italy and LASE-Lab. of Advanced Solar Energy, National University of Science and Technology 'MISiS', Moscow, Russia*

E-mail: rosanna.larciprete@isc.cnr.it; antonio.agresti@uniroma2.it; pescetel@uniroma2.it

Atomic photoionization cross sections

Table S 1: Calculated atomic cross sections for photoionization taken from Ref.[1]

photon energy (eV)	I3d	O1s	N1s	C1s	Pb4f	Cs4d	Br3d	I4d
250					1.7	0.89	4.24	0.87
520			0.39	0.24	3.87	0.60	0.89	0.51
1000	1.30	0.11	0.07	0.04	1.00	0.18	0.14	0.15

Fresh samples

X-ray induced core level shifts Fig.S1 shows the shifts in binding energy of the $Pb4f_{7/2}$ and $I4d_{5/2}$ peaks observed in the PV sample exposed to the X-ray beam.

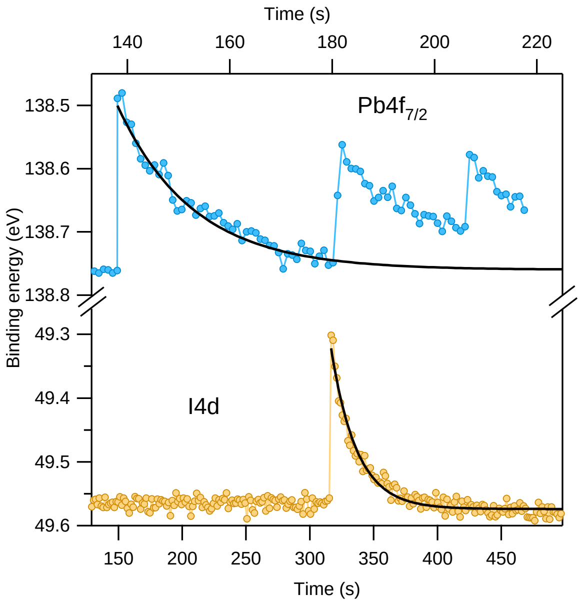

Fig.S 1: $Pb4f_{7/2}$ and $I4d_{5/2}$ binding energy vs. time for the 2D maps shown in Fig.1. The black lines represent the best-fit curves of the experimental data by using the relation $BE = BE_0 - A \times exp[(t_0 - t)/\tau]$ for $t \geq t_0$. The resulting τ values were 16.4 and 20.1 s, for Pb4f and I4d, respectively. The photon energy was 520 eV and the photon flux was the same in the two cases.

Chemical composition The core level intensities for the different elements measured for PV and PVMX samples, in the various aging stages, are reported in Table S2. Fig.S2 compares the XPS survey spectra measured at photon energy of 520 eV for dark-aged and light-aged PV and PVMX samples.

Table S 2: Pb4f, I4d and Br3d integrated intensities, corrected for the photoionization cross sections (see Table.SI) and normalized to the Pb4f intensity, obtained for PV and PVMX from the bulk sensitive ($h\nu$=1000 eV) and surface sensitive ($h\nu$=250 eV) survey spectra shown in Figures 2 and 4. The right column lists in each case the corresponding $Pb(I_xBr_y)_3$ composition. The error bar on x and y is ±5%

state	$h\nu$ (eV)	sample	Pb4f	I4d	Br3d	$Pb(I_xB_y)_3$
fresh	250	PV	1	2.02	0.57	$Pb(I_{0.67}Br_{0.19})_3$
	250	PVMX	1	2.46	0.47	$Pb(I_{0.82}Br_{0.16})_3$
	1000	PV	1	2.69	0.39	$Pb(I_{0.89}Br_{0.13})_3$
	1000	PVMX	1	2.44	0.43	$Pb(I_{0.81}Br_{0.14})_3$
dark-aged	250	PV	1	1.66	0.61	$Pb(I_{0.55}Br_{0.20})_3$
	250	PVMX	1	1.88	0.60	$Pb(I_{0.63}Br_{0.20})_3$
	1000	PV	1	2.70	0.46	$Pb(I_{0.90}Br_{0.15})_3$
	1000	PVMX	1	2.95	0.49	$Pb(I_{0.98}Br_{0.16})_3$
light-aged	250	PV	1	0.56	0.56	$Pb(I_{0.19}Br_{0.19})_3$
	250	PVMX	1	0.56	0.54	$Pb(I_{0.19}Br_{0.18})_3$
	1000	PV	1	0.58	0.53	$Pb(I_{0.19}Br_{0.18})_3$
	1000	PVMX	1	0.62	0.51	$Pb(I_{0.21}Br_{0.17})_3$

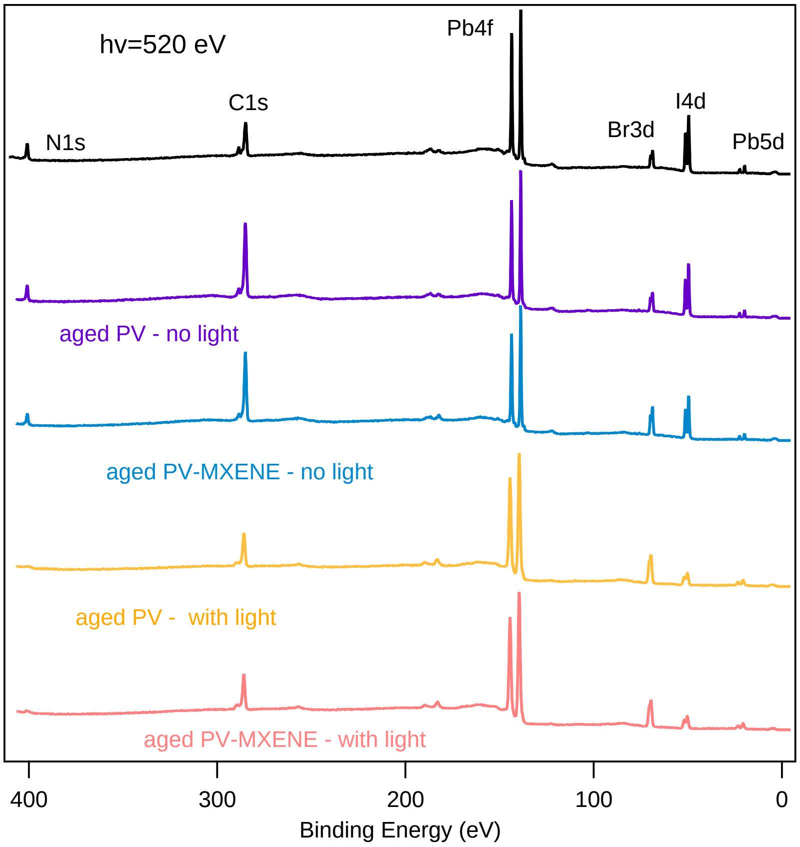

Fig.S 2: Survey spectra measured with photon energy of 520 eV on PV and PVMX samples kept for 30 days in atmosphere in the dark ("dark-aged") or also exposed to 1-sun radiation ("light-aged"). The corresponding spectrum measured on a fresh PV layer is shown for comparison at the top of the panels.

Valence band In the valence band spectra shown in Fig.2h the main spectroscopic features A-X have been modelled for $MAPbBr_3$ and $MaPbI_3$, as well as for the mixed perovskite $(FA_{0.83}MA_{0.17})Pb(I_{0.83}Br_{0.13})_3$ by DFT calculations in Ref.[2] Briefly the A and B features are assigned to mixed Pb-halogen states, with the Pb-I band located at the top of the valence band. Halogen-Pb mixed states originate also the D (I-Pb and Br-Pb) and F (I-Pb) bands, whereas C (C2p state) and E (N2p state) derive from the organic phase. The main difference between the PV and PVMX spectra arises in the 7-9 eV region, in correspondence of the C band, where the stronger intensity for PVMX has to be related to the higher intensity of the contaminating peak in the C1s spectrum. The same A-F features appear in the VB

measured at 520 eV on the PV sample, the line shape difference with respect to the 100 eV spectrum being due partly to the composition gradient along the layer thickness discussed above and partly to the ionization cross sections of the different bands at the two photon energies.[2]

Light-aged samples

Analysis of the core level spectra Here we discuss the results of the decomposition of the high resolution core level spectra measured on the light-aged samples and shown in Fig.8 of the main text. For the $Pb4f_{7/2}$, Br3d and I4d spectra we found some residual intensity of the fresh PV spectra (components Pb_A, I_A, Br_A) and, in each case, intense new components (Pb_B, I_B, Br_B) shifted by 600-650 meV to higher BE. As observed in Figures 5 and 6, the light-aged samples manifest heavy depletion of iodine and strong loss of N with clear signs of MA and FA decomposition. For $MAPbI_3$, I and MA vacancies are reported to cause intrinsic doping of the opposite sign. By assuming a similar trend also in mixed perovskites, the observed blue-shift could indicate that for the light-aged samples the resulting effect is a net n-doping with the photoelectron spectra aligning with the new position of the Fermi level, which approaches the vacuum level. However, in both PV and PVMX samples also the whole C1s spectra, which mostly arises from surface contaminants, moves to the high BE by an even higher quantity. Moreover, by comparing the spectra measured at different photon energies in the VB region (see Fig.S3) it turns out that the spectrum measured at 100 eV moves higher in BE with respect to those measured with 250-1000 eV photons, which instead are all nearly superimposed. At a first glance this behavior could be attributed to a heavier n-doping effect at the surface layer, due to a more severe degradation of the perovskite matrix compared to the bulk. Indeed, the sample surface is optimally probed with the reduced kinetic energy (85-96 eV) of the electron photoemitted at $h\nu$=100 eV. However, the C1s, and $Pb4f_{7/2}$ spectra, when measured at comparably low kinetic energy

(105-110 eV) do not manifest any extra-shift with respect to the spectra measured with higher photon energy (see Fig.S3).

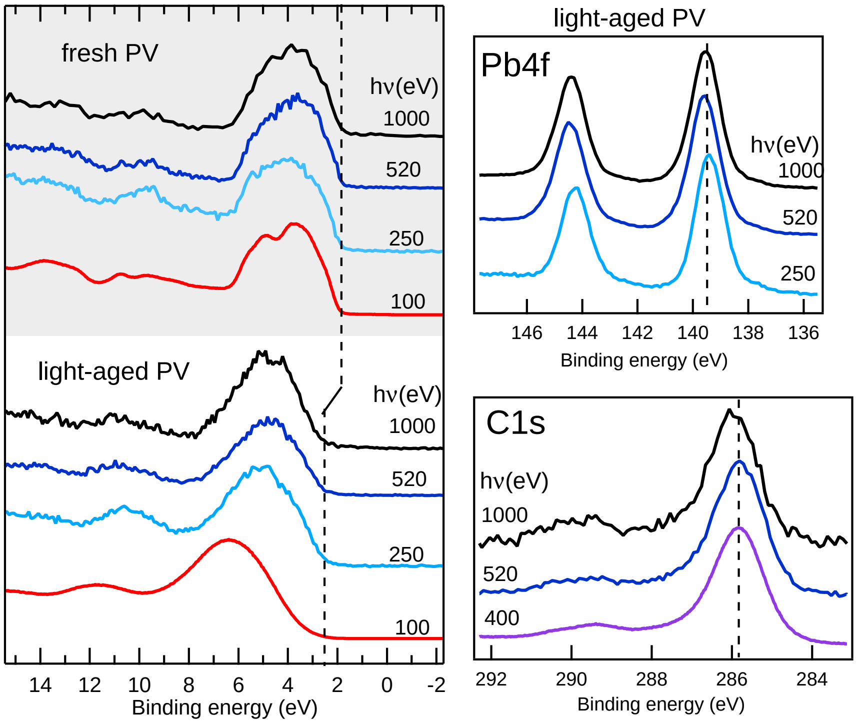

Fig.S 3: left) Valence band spectra measured on fresh and light-aged PV at different photon energies. In the case of the fresh sample the VBM is located at the same binding energy in all spectra independently on the photon energy. For the aged sample, in the spectra measured with photon energy $\geq$ 250 eV the VB maxima are coincident with each other and are shifted by $\sim$ 800 meV with respect to the fresh sample. Differently, the spectrum measured at hν=100 eV shows an extra shift of about -1.2 eV. Right) Pb4f and C1s core level spectra measured on the light aged sample at different photon energies. In this case the spectra are all superimposed. See the main text for details

Then there should be an additional factor causing the extra-shift. A possible way to rationalize the observed behavior, which resembles a much more intense manifestation of the photon induced effect illustrated in Fig.1, is to assume that in the degraded perovskite matrix the x-ray beam induces a photogenerated electric field, which determines the blue-shift of the spectral features. This effect, which cannot be clearly disentangled from the severe

chemical degradation and the possible presence of doping, becomes dramatically stronger at low photon energy.

Assignment of the Pb4f spectrum Compounds commonly found in decomposed perovskites are PbO and $PbCO_3$.[3–5] The first one is distinguished by $Pb4f_{7/2}$ and O1s components at 137.8-138.6 eV[4,6] and 529.3 - 529.7 eV,[4,6] respectively, whereas for $PbCO_3$ the $Pb4f_{7/2}$, O1s and C1s peaks are expected in order at $\sim$ 139.3, $\sim$ 531.1 and $\sim$ 289 eV.[4] As shown in Fig.8, in addition to Pb_A (138.76 eV) and Pb_B (139.41 eV), the $Pb4f_{7/2}$ spectrum exhibits a weak component at 138.04 and a more intense component at 139.87 eV. When corrected for the ~650 meV shift, the locations of the two latter peaks get close to the BEs expected for PbO and $PbCO_3$.

$Ti_3C_2T_x$ work function

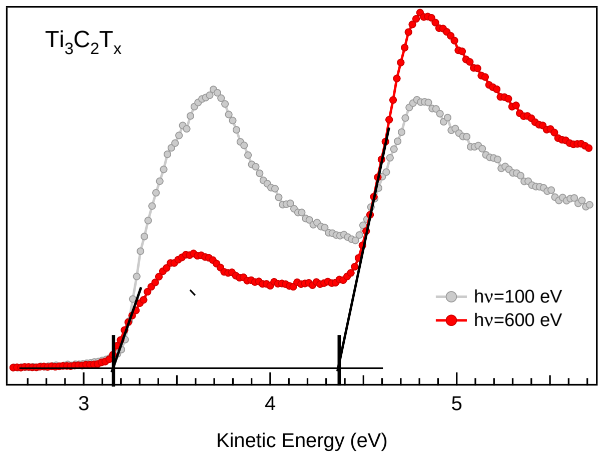

Fig.S 4: Secondary electron cutoff measured for a $Ti_3C_2T_x$ MXene layer at photon energy of 100 eV (surface sensitive) and 600 eV (bulk sensitive). The energy shift between the two edges can be related to the presence of the oxidized phases located mostly at the sample surface and much less in the layers underneath. The MXenes flakes were mixed to the PV precursors immediately after their synthesis whereas these WF measurements were carried out a few days after. Therefore the WF value measured on the less oxidized bulk is reasonably representative of the as as prepared $Ti_3C_2Ti_x$ flakes.

Thermal stress

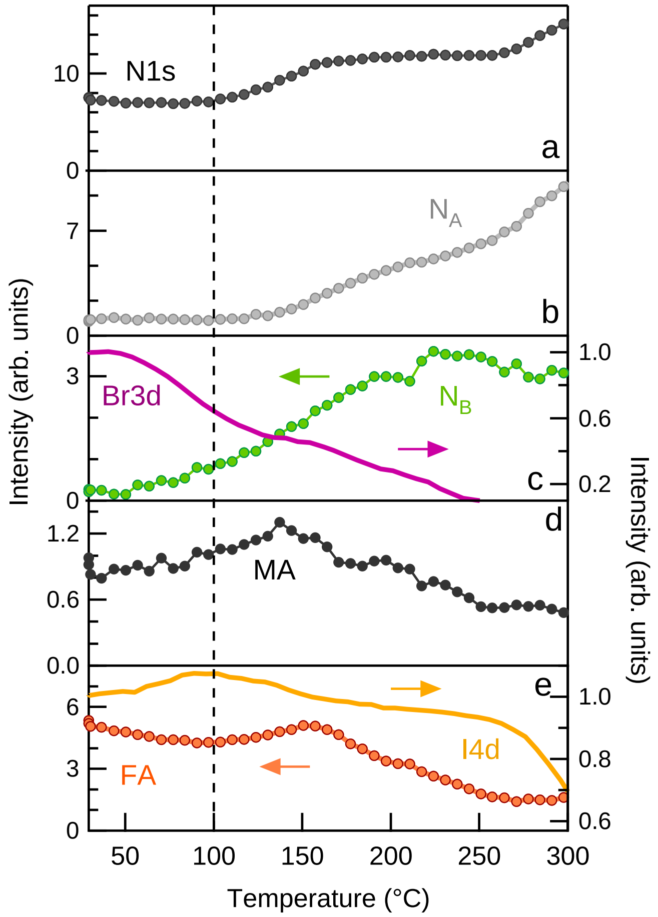

Fig.S 5: Photoemission intensity vs. temperature plotted for the core level spectra measured during the annealing of the PV sample (compare Fig.10a). Panel a) shows the intensity of the whole N1s spectrum; panels b-e) display the intensities of the N1s spectral components N_B, MA and FA in expanded scales with respect to Fig.10a. Panels c) and e) show also the intensities of the Br3d and I4d spectra, respectively, conveniently expanded (refer to the right axes) to be optimally compared with the N_B and FA curves.

References

1. Yeh, J. *Atomic Calculation of Photoionization Cross-Sections and Asymmetry Parameters*; Gordon and Breach Science Publishers, Langhorne, PE (USA), 1993.

2. Philippe, B.; Jacobsson, T. J.; Correa-Baena, J.-P.; Jena, N. K.; Banerjee, A.;

Chakraborty, S.; Cappel, U. B.; Ahuja, R.; Hagfeldt, A.; Odelius, M.; Rensmo, H. Valence Level Character in a Mixed Perovskite Material and Determination of the Valence Band Maximum from Photoelectron Spectroscopy: Variation with Photon Energy. *J. Phys. Chem C* **2017**, *121*, 26655–26666.

3. Climent-Pascual, E.; Hames, B. C.; Moreno-Ramírez, J. S.; Álvarez, A. L.; Juarez-Perez, E. J.; Mas-Marza, E.; Mora-Seró, I.; de Andrés, A.; Coya, C. Influence of the substrate on the bulk properties of hybrid lead halide perovskite films. *J. Mater. Chem. A* **2016**, *4*, 18153–18163.

4. Huang, W.; Manser, J. S.; Kamat, P. V.; Ptasinska, S. Evolution of Chemical Composition, Morphology, and Photovoltaic Efficiency of $CH_3NH_3PbI_3$ Perovskite under Ambient Conditions. *Chem. Mater.* **2016**, *28*, 303–311.

5. Rocks, C.; Svrcek, V.; Maguire, P.; Mariotti, D. Understanding surface chemistry during $MAPbI_3$ spray deposition and its effect on photovoltaic performance. *J. Mater. Chem. C* **2017**, *5*, 902–916.

6. Terpstra, H. J.; de Groot, R. A.; Haas, C. Electronic structure of the lead monoxides: Band-structure calculations and photoelectron spectra. *Phys. Rev. B* **1995**, *52*, 11690–11697.
